# Design and implementation of a low-power position monitoring and control system for on-orbit focusing

**DOI:** 10.1371/journal.pone.0330026

**Published:** 2025-08-08

**Authors:** Leijie Jiang, Keyu Guo, Xin Liu, Zhenzhong Zhang

**Affiliations:** 1 School of Mechanical Engineering and Rail Transit, Changzhou University, Changzhou, China; 2 Jiangsu Key Laboratory of Green Process Equipment, Changzhou University, Changzhou, China; 3 Tianshui Electric Drive Research Institute Group Co., Ltd., Tianshui, China; IIIT Kurnool: Indian Institute of Information Technology Design and Manufacturing Kurnool, INDIA

## Abstract

To enable the optical remote sensing satellite to flexibly adjust the camera’s focal length for clearer imaging, a low-power position monitoring and control system suitable for space environments is designed, which is highly significant for aerospace devices with limited energy resources. A novel constant voltage drive circuit is proposed for driving the stepper motor. The differential linear driving and receiving circuit is presented to collect data from the linear encoder. By using the linear encoder to provide feedback on the current focal length, both open-loop and closed-loop focusing functions are achieved. The performances of the designed system are implemented on a field programmable gate array (FPGA) circuit board. The experimental results demonstrate that the developed system consumes approximately 1.4 W of power, has a strong load-carrying capacity, and achieves a focusing control precision of ±0.0004 mm over the entire motion range of −6 mm to +6 mm.

## Introduction

The camera, a payload on the optical remote sensing satellite, photographs Earth’s landscape. The resulting image data are crucial for accurate analysis in diverse fields such as agriculture, forestry, marine science, land management, environmental protection, and meteorology. Image sharpness, which is essential for data accuracy, depends on precise camera focus. This focus is adjusted by the focusing mechanism within the satellite’s camera subsystem.

The focusing technology has been developed for many years, the optical device aperture and optical system structure used in focusing mechanism have shown a diversified development trend [1–4]. The focusing mechanism extends from one-dimensional directional control to multi-dimensional spatial control. Due to the application of various new technologies in the focusing mechanism, the focusing mechanism has a significant development in the simplification of the structure, high reliability, space adaptability, focusing speed and accuracy, level of intelligence, power consumption, interference resistance, and integration [5–10]. Aiming at the problem that the alignment between primary mirror and secondary mirror in the optical system of small satellite may be affected by the harsh environment of spacecraft and space, a flexible hinge focusing mechanism driven by a single motor is proposed in [11]. An autonomous focusing mechanism is designed to adjust lens displacement to achieve the best focusing position through image contrast analysis [12]. A high-precision programmable focusing mechanism (PFM) has been developed and designed for the use in vision systems in semiconductor devices [13]. A focusing mechanism based on the optimal focusing of a space carbon dioxide analyser is designed in [14]. Three piezoelectric drive motors are employed to achieve three-dimensional adjustment of the detector. The detector is placed in the optimum optical imaging position by the feedback from a displacement sensor and the thickness of the mounting spacer is adjusted to suit the motor drive. A novel mechanism is presented to reduce the operating temperature of a thermally driven refocusing mechanism [15]. In contrast to thermally driven systems, a refocusing mechanism based on direct transmission is proposed in [16]. The secondary mirror is controlled by linear motion, and the tilt is achieved by controlling rotational motion. However, by using two actuators, the energy consumption of such a mechanism can be quite high, compared to a mechanism driven by a single actuator.

With the development and progress of micro science and technology, the auto-focusing microscope is replacing manual focusing microscope in more and more fields. The reason is that the auto-focusing system realizes the automation of the imaging process. In addition, it is able to reduce the effect of the focusing errors imposed by manual operation. Typical applications include robotic cell micromanipulation and imaging [17–19], scanning probe microscopy (SPM) and nanolithography [20,21], a broader range of microscopes [22], detailed microscopic imaging [23], complex imaging environments [24], and an adaptive liquid lens microscope system [25]. Currently, some satellite-borne focusing devices are driven by a DC motor [11], however, the positioning accuracy of such control systems is typically not very high, making it difficult to meet the demands for high-precision focusing. Some satellite-borne focusing devices are driven by a screwed-type piezoelectric actuator [26], however, the high-precision control of such systems is relatively complex, and the output torque is usually not very high, making it difficult to meet the application requirements for high power output. Some focusing mechanism drive systems are not suitable for use in harsh working environments [11,26,27]. Moreover, the drive systems in [11,26,27] exhibit relatively low levels of integration and scalability.

From previous research, it is clear that the existing control systems for focusing mechanisms are either only suitable for operation in a good ground-based environment, such as a narrow temperature variation range and weak cosmic high-energy particle radiation, or they lack sufficient control precision, or they employ relatively complex high-precision control strategies and they have inadequate driving capability. Furthermore, their integration and scalability are relatively poor. However, the proposed focusing mechanism is located in a harsh aerospace environment containing the wide temperature variation range and complex radiation from gamma rays, high-energy protons, and cosmic rays, which can lead to the failure of low-quality electronic components and single event upset (SEU) of the digital signal processor (DSP) or other microcontroller unit (MCU) devices. This can result in loss of measurement data and interruption of the system’s functionality. Furthermore, the heavy satellite-borne focusing mechanism needs to be precisely controlled. As a result, these previous systems cannot fulfil the requirements. It is necessary to develop a novel control system employing high-grade, anti-irradiation electronic components to ensure superior anti-irradiation performance, drive capability, control precision, and high levels of integration and scalability.

The main contribution of this work is the design of a low-power position monitoring and control system with high positioning accuracy and strong driving capability for the automatic focusing of the satellite-borne focusing mechanism. In this paper, a focusing mechanism position monitoring and control system for the aerospace environment is designed and implemented. FPGA is chosen to parse the C-mode bidirectional serial synchronous (BiSS) protocol of the linear encoder from Renishaw in order to obtain the feedback data. In addition, after receiving the commands from the host computer, FPGA controls the rotation of the stepper motor through the motor drive circuit to realize the open-loop focusing and closed-loop focusing functions.

## System hardware circuit design

### Overall design of the system hardware

**Fig 1:**
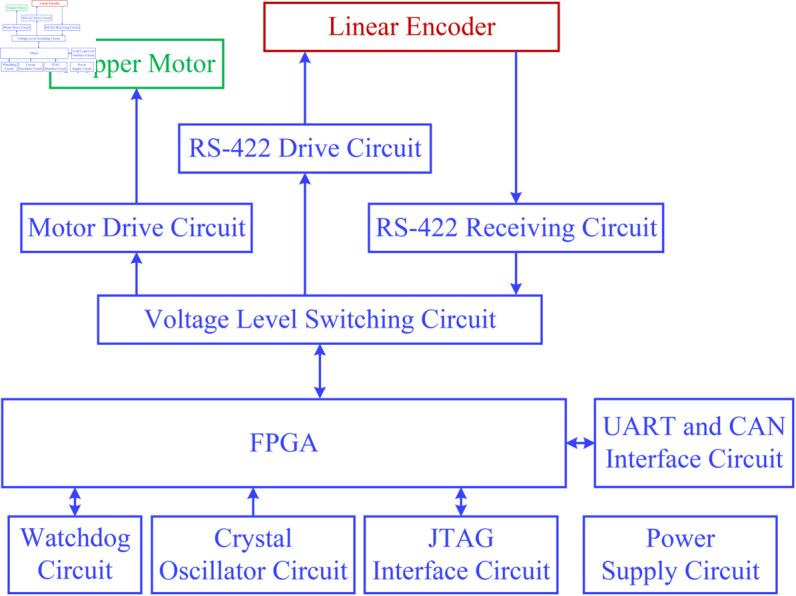
Block diagram of the system hardware design.

The block diagram of the system hardware design is shown in Fig 1. The motor drive circuit is designed to convert weak electrical control signals into amplified electrical signals for driving the stepper motor. The position acquisition requests and timing information (clock) are transmitted from FPGA to the linear encoder through the designed recommended standard 422 (RS-422) drive circuit. The position data is transferred from the linear encoder to FPGA through the designed RS-422 receiving circuit. The watchdog circuit is employed to monitor the running process of FPGA. The crystal oscillator circuit can generate the 50-MHz clock for FPGA. The host computer with controller area network (CAN) debugging assistant software or the serial debugging assistant software communicates with FPGA via CAN or universal asynchronous receiver/transmitter (UART) interface. Each circuit is powered by the power supply circuit. Next, the motor drive circuit will be introduced.

### Design of the motor drive circuit

**Fig 2:**
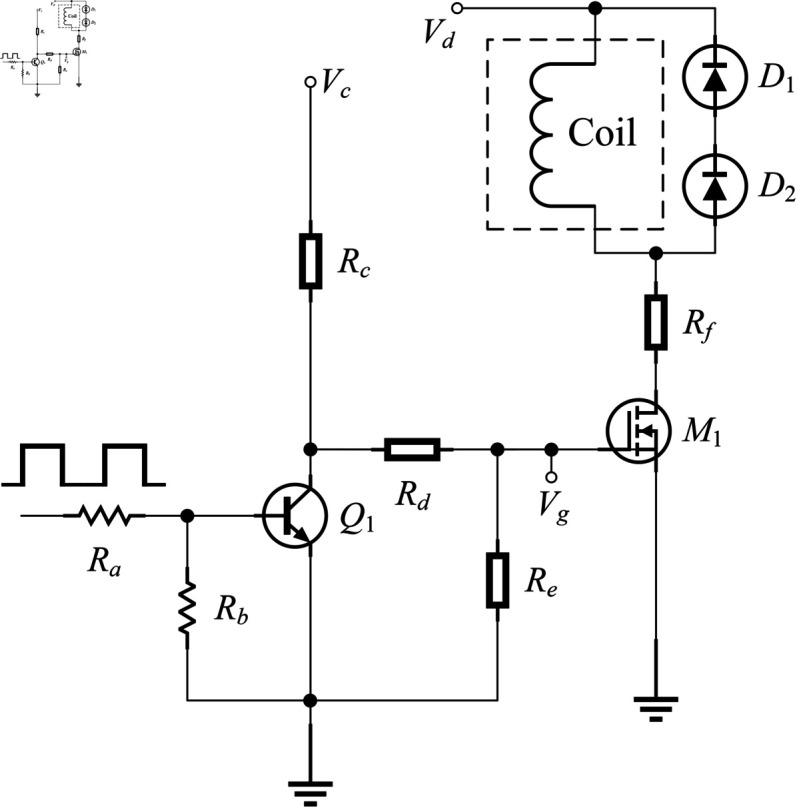
One-phase drive circuit schematic for the unipolar stepper motor.

The good motor drive circuit is crucial for realizing the accurate stepper motor control. Fig 2 designs one-phase drive circuit for the unipolar stepper motor. It is used to elaborate on the design process of the drive circuit and its working principle. An *m*-phase stepper motor has *m* phases, each typically with one coil, resulting in a total of *m* coils. It’s important to note that “coil” in Fig 2 represents only one phase of the motor, not the entire motor itself. The *m*-phase stepper motor will require *m* such one-phase drive circuits. *R*_*a*_, and *R*_*b*_ are the surface mount device (SMD) resistors (kΩ), *R*_*c*_, *R*_*d*_, and *R*_*e*_ are the through-hole resistors (kΩ), *R*_*f*_ is the wirewound resistor (Ω), *Q*_1_ is a transistor, *D*_1_, and *D*_2_ are the switching diodes, *M*_1_ is a power MOSFET, $V_{g}$ is the gate potential of *M*_1_ (V), $V_{c}$ is the voltage of power supply of the control circuit (V), $V_{d}$ is the voltage of power supply of the drive circuit (V), the gate threshold voltage of *M*_1_ is denoted as $V_{th1}$ (V), the collector-to-emitter on-state resistance of *Q*_1_ is denoted as *R*_*CE*_ (kΩ), the phase inductance is denoted as *L* (H), the current flowing through *R*_*f*_ is denoted as *I*_*R*_ (A), the drain-to-source breakdown voltage of *M*_1_ is denoted as $V_{BV}$ (V).

When the low level arrives, *Q*_1_ is in the off state. In this case, the working loop for $V_{c}$ is $V_{c}\to R_{c}\to R_{d}\to R_{e}\to \text{GND}\to V_{c}$. Therefore, one obtains

$$V_{g}=V_{c}\frac{R_{e}}{R_{c}+R_{d}+R_{e}}$$ (1)

The on-state condition of the MOSFET is that the gate voltage needs to exceed the threshold voltage. For the negative-MOSFETs (N-MOSFETs), in order to keep *M*_1_ in the on state, one has

$$V_{g}>V_{th1}$$ (2)

Based on Eqs (1), and (2), one has

$$V_{c}\frac{R_{e}}{R_{c}+R_{d}+R_{e}}>V_{th1}$$ (3)

Eq (3) is the selection condition for *R*_*c*_, *R*_*d*_, and *R*_*e*_. In order to limit the operating current, they are usually selected as resistance values in the kilohm class.

When the high level arrives, *Q*_1_ is in the on state. In this case, since *R*_*CE*_ is very small, the working loop for $V_{c}$ is $V_{c}\to R_{c}\to Q_{1}\to \text{GND}\to V_{c}$. It can be deduced that $V_{g}\approx 0$. Hence, one obtains $V_{g}<V_{th1}$. Under this condition, *M*_1_ is in the off state.

When *M*_1_ transitions from the on state to the off state, *I*_*R*_ will inevitably decrease. Therefore, the coil will generate an induced electromotive force in a downward direction *E*_*z*_ (V). If *E*_*z*_ is not less than $V_{BV}$, *M*_1_ will be broken down in the absence of *D*_1_ and *D*_2_. In the presence of *D*_1_ and *D*_2_, *E*_*z*_ will have a current continuation loop $E_{z}\to D_{2}\to D_{1}\to E_{z}$. After the current continuation, *E*_*z*_ will be less than $V_{BV}$. This will protect *M*_1_ well against the breakdown.

When *M*_1_ transitions from the off state to the on state, *I*_*R*_ will inevitably increase. Hence, the coil will generate an induced electromotive force in an upward direction *E*_*f*_ (V). Since *D*_1_ and *D*_2_ have the unidirectional conduction characteristics, *E*_*f*_ cannot have an effect on *M*_1_. This will also protect *M*_1_ against the breakdown.

The time constant of current rise can be expressed as

$$T_{c}=\frac{L}{R_{f}}$$ (4)

Eq (4) is an important basis for selecting *R*_*f*_. If the output torque of the motor is to be increased, *T*_*c*_ should be reduced to steepen the rising edge of *I*_*R*_. After the motor is manufactured, *L* is usually a constant value. Therefore, a common practice is to increase *R*_*f*_ in order to decrease *T*_*c*_. In short, the increase of *R*_*f*_ makes the waveform of *I*_*R*_ more rectangular. In this way, the output torque of the motor can be increased, as well as the startup and continuous operation frequency of the motor can be improved. In theory, maintaining the same steady-state current requires a corresponding increase in $V_{d}$. However, in practice, $V_{d}$ is often a fixed value and cannot be adjusted. Therefore, *R*_*f*_ cannot be increased indefinitely. Extensive experimentation has shown that selecting a resistance value in the ohms class typically achieves satisfactory results.

After receiving a set of control commands with the high and low levels, *m* such one-phase drive circuits drive the stepper motor by continuously switching on and off the N-MOSFETs. The designed motor drive circuit uses only one control voltage $V_{c}$ and one drive voltage $V_{d}$. It has the simple circuits, few power amplifier components, and the low cost.

### Design of the RS-422 transceiver circuit

**Fig 3:**
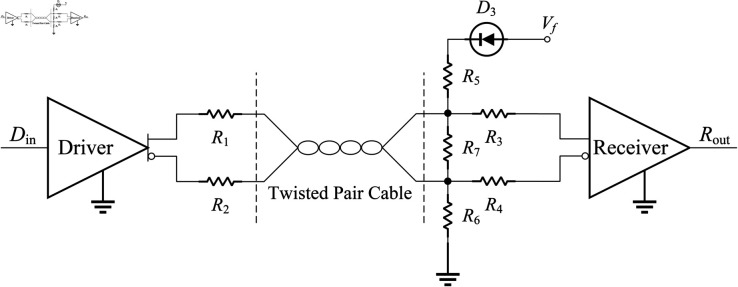
RS-422 transceiver circuit schematic.

The electrical specification of the hardware interface between FPGA and the linear encoder from Renishaw follows the RS-422 specification [28]. The communication between FPGA and linear encoder belongs to one-to-one transceiver form. A one-to-one RS-422 transceiver circuit is presented in Fig 3. FPGA and the linear encoder are mutual transceivers. $D_{\text{in}}$ is the signal from FPGA or the linear encoder, and $R_{\text{out}}$ is the signal received by the linear encoder or FPGA, *D*_3_ is a diode used to prevent power supply backflow, $V_{f}$ is the voltage of the power supply for the RS-422 transceiver circuit, *R*_1_, *R*_2_, *R*_3_, *R*_4_, *R*_5_, *R*_6_, and *R*_7_ are the SMD resistors. *R*_1_ and *R*_2_ are recommended to be 50 Ω, *R*_3_ and *R*_4_ are recommended to be 1000 Ω, *R*_5_ and *R*_6_ are recommended to be 750 Ω, *R*_7_ is recommended to be 130 Ω. These recommended resistor values follow industry standards and are derived from extensive experiments and parameter optimization. Furthermore, in aerospace design, $V_{f}$ is typically taken as $V_{f}=5$ V.

The brief design of the motor drive circuit and RS-422 transceiver circuit has been described. Next, the detailed design of this circuit will be presented.

### Detailed design of the motor drive circuit and RS-422 transceiver circuit

**Fig 4:**
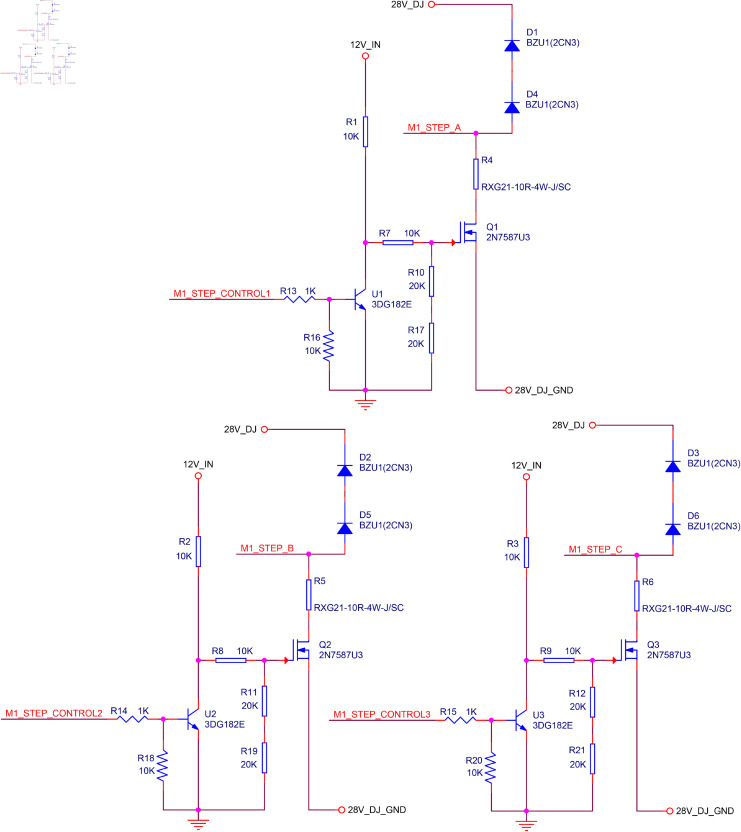
Constant voltage drive circuit of the stepper motor.

**Fig 5:**
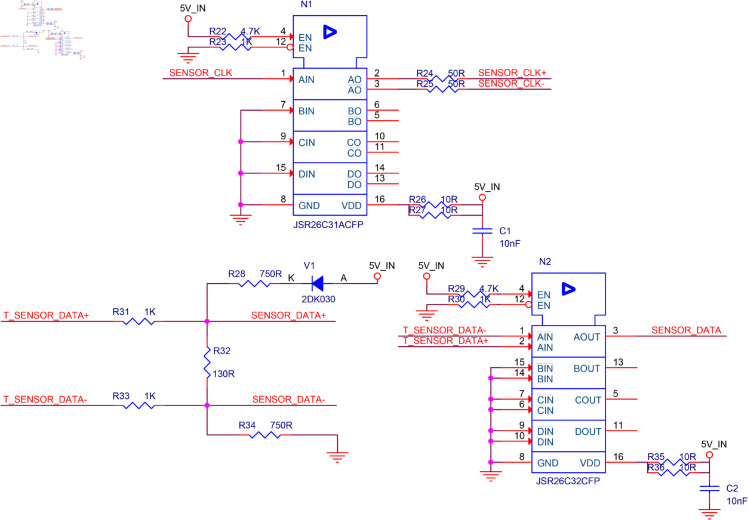
RS-422 transceiver circuit.

In Figs 4 and 5, the constant voltage drive circuit of the stepper motor and RS-422 transceiver circuit are designed by using Cadence software. To consider the light weight and small size of the system hardware, the types of the resistors and capacitors used in the circuit have been distinguished to provide a reference for the correlative circuit design.

Finally, the main work done in the design of these two circuits is summarized as follows:

- The selection criteria for *R*_*c*_, *R*_*d*_, and *R*_*e*_ are presented in Eq (3).
- The selection criterion for *R*_*f*_ is presented in Eq (4).
- There are no specific selection criteria for *R*_*a*_ and *R*_*b*_. To reduce current and power consumption, these resistor values are typically chosen to be in the kilo-ohm range. The recommended values are generally *R*_*a*_ = 1 kΩ and *R*_*b*_ = 10 kΩ. Their parameters are usually determined based on the debugging method.
- In Fig 3, the recommended values for each resistor obtained through debugging methods are provided.

The focusing method of the mechanism will be introduced in the following section.

## Focusing method of the mechanism

### Relationship between the focusing position and the number of motor steps

**Fig 6:**
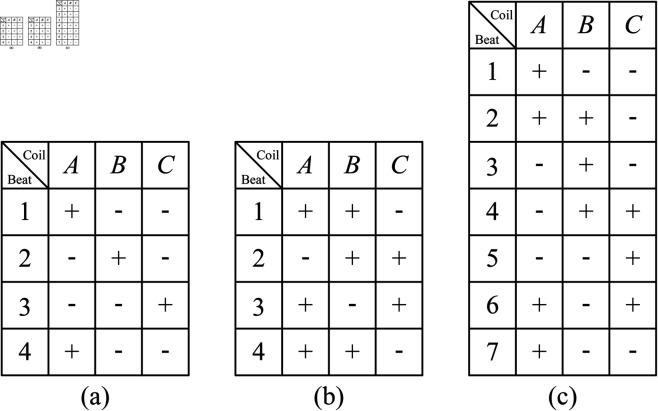
Common drive modes of the stepper motor. (a) Single triple beat. (b) Double triple beat. (c) Single and double six-beat.

The focusing mechanism mainly consists of the stepper motor, the designed drive system, the reducer, the ball screw, and the linear encoder. In order to be able to focus accurately, the relationship between the focusing position and the number of steps of the stepper motor needs to be obtained. In Fig 6, “ + ” indicates that the coil is powered on and “–” indicates that the coil is powered off. When the stepper motor operates in single triple beat mode or double triple beat mode, its step angle is $1.8^{\circ }$. When the stepper motor operates in single and double six-beat mode, its step angle is $0.9^{\circ }$. If the angular velocity of the motor is denoted as $\omega_{m}$ (rad/s), the output angular velocity of the reducer is denoted as $\omega_{r}$ (rad/s), the running time of the motor is denoted as *t*_*r*_ (s), and the gear ratio of the reducer is denoted as *i*. In time *t*_*r*_, the motor completes *r*_*m*_ revolutions and the screw completes *r*_*s*_ revolutions. Based on these parameters, one obtains

i=ωmωr=ωmtrωrtr=ωmtr/ωmtr2π2πωrtr/ωrtr2π2π=rmrs (5)

Based on Eq (5), when *r*_*s*_ = 1, *r*_*m*_ = *i*. The lead of the ball screw is denoted as *L*_*l*_ (mm). Since *L*_*l*_ refers to the linear distance the nut moves along the axis when the screw makes one full rotation, it can be concluded that when the motor completes *i* revolutions, the linear distance the nut moves along the axis is *L*_*l*_. In the proposed scheme, the motor operates in double triple beat mode (step angle is $1.8^{\circ }$). Therefore, the motor needs to take 200 steps to complete one full rotation. If the straight-line movement distance of the nut corresponding to each step of the motor is denoted as *L*_*s*_ (mm), based on these parameters, one has

$$200iL_{s}=L_{l}$$ (6)

Based on Eq (6), one obtains

$$L_{s}=\frac{L_{l}}{200i}$$ (7)

If the desired focusing position is denoted as *S*_*d*_, and the number of motor steps corresponding to the desired focusing position is denoted as *N*_*d*_, by Eq (7), one has

$$N_{d}=\frac{S_{d}}{L_{s}}=\frac{200iS_{d}}{L_{l}}$$ (8)

### Measurement of the focusing position

The accurate measurement of the focusing position is essential for the focusing. Whether closed-loop or open-loop focusing, they are very dependent on the real-time focusing position. The linear encoder from Renishaw is employed to measure the focusing position. If the grid pitch of the linear encoder is denoted as *l*_*g*_ (mm), and the 26-bit digital signal read by FPGA is denoted as *D*_*s*_ (decimal value), one has

$$S_{c}=l_{g}D_{s}$$ (9)

where *S*_*c*_ is the current focusing position corresponding to *D*_*s*_.

Based on Eqs (7)–(9), if the current focusing position and the desired focusing position are located on one side of the 0-point position, the number of motor steps corresponding to the distance between them can be expressed as

$$N_{d1}=\frac{\left|S_{d}-S_{c}\right|}{L_{s}}=\frac{200i\left|S_{d}-S_{c}\right|}{L_{l}}=\frac{200i\left|S_{d}-l_{g}D_{s}\right|}{L_{l}}$$ (10)

Based on Eqs (7)–(9), if the current focusing position and the desired focusing position are located on either side of the 0-point position, the number of motor steps corresponding to the distance between them can be expressed as

$$N_{d2}=\frac{\left|S_{d}+S_{c}\right|}{L_{s}}=\frac{200i\left|S_{d}+S_{c}\right|}{L_{l}}=\frac{200i\left|S_{d}+l_{g}D_{s}\right|}{L_{l}}$$ (11)

### Open-loop and closed-loop focusing

**Fig 7:**
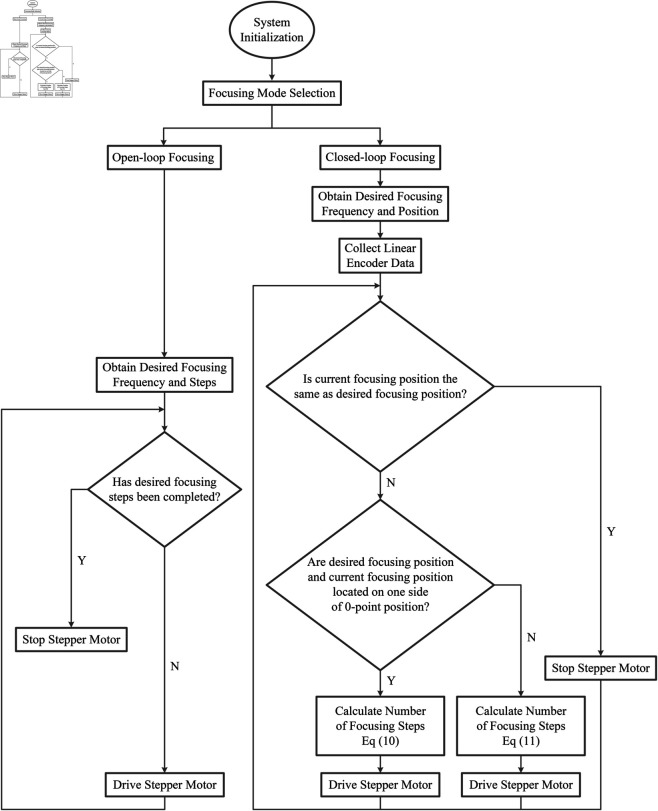
Flowchart of the focusing software.

The software algorithm is executed to flexibly adjust the focal length of the camera for acquiring clearer image data according to the flowchart shown in Fig 7. Fig 7 presents two strategies: open-loop focusing and closed-loop focusing. For open-loop focusing, the program generates driving signals with corresponding frequency and pulse count based on the focusing frequency and steps specified in the open-loop focusing instructions, using an FPGA. These signals, along with other control signals, are applied to the driver chip to achieve the open-loop focusing function. For closed-loop focusing, the program sends data acquisition instructions to the linear encoder, which then provides the feedback on the current focusing position. Based on the difference between the desired focusing position and the current focusing position, the focusing steps are calculated. Corresponding driving signals are generated based on the specified frequency, and these signals, along with other control signals, are applied to the driver chip to achieve the closed-loop focusing function. Generally, open-loop focusing is chosen for coarse focusing, while closed-loop focusing is selected for fine focusing. The analysis of the system’s load-carrying capacity will be presented in the following section.

## Load-carrying capacity of the system

### Output torque of the motor

If the output power of the position monitoring and control system is denoted as $P_{\text{in}}$ (W), the efficiency of the motor is denoted as η, and the output power of the motor is denoted as $P_{\text{out}}$ (W), one has

$$\eta =\frac{P_{\text{out}}}{P_{\text{in}}}$$ (12)

If the output torque of the motor is denoted as *T*_*m*_ (N·m), one obtains

$$P_{\text{out}}=T_{m}\omega_{m}$$ (13)

If the speed of the motor is denoted as *n*_*m*_ (r/s), one has

$$\omega_{m}=2\pi n_{m}$$ (14)

Based on Eqs (12)–(14), one obtains

$$T_{m}=\frac{\eta P_{\text{in}}}{2\pi n_{m}}$$ (15)

### Output torque of the reducer

If the output power of the reducer is denoted as *P*_*r*_ (W), and the output torque of the reducer is denoted as *T*_*r*_ (N·m), one obtains

$$P_{r}=T_{r}\omega_{r}$$ (16)

Because the input power and output power of the reducer are nearly equal, one has

$$P_{\text{out}}\approx P_{r}$$ (17)

Based on Eqs (13), (16), and (17), one obtains

$$T_{r}\approx T_{m}\frac{\omega_{m}}{\omega_{r}}$$ (18)

Based on Eqs (5), (15), and (18), one has

$$T_{r}\approx \frac{\eta P_{\text{in}}}{2\pi n_{m}}i$$ (19)

From Eq (19), when $P_{\text{in}}$ increases and *n*_*m*_ decreases, *T*_*r*_ increases. Due to the designed position monitoring and control system’s ability to output a large power, it has strong load-carrying capacity at low speeds. The implementation of a position monitoring and control system of satellite-borne focusing mechanism will be described in the following section.

## System implementation

### Experimental system configuration

**Fig 8:**
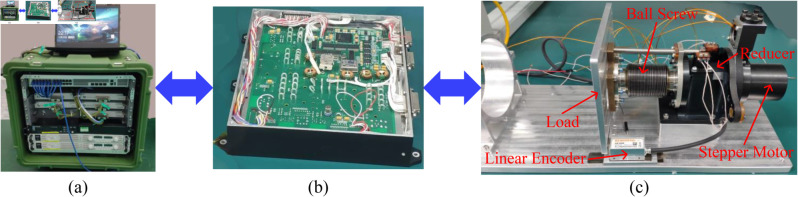
Experimental setup: (a) ground test equipment, (b) position monitoring and control system hardware, (c) focusing mechanism.

Fig 8 shows the experimental setup. It mainly consists of the ground test equipment, the position monitoring and control system hardware, and the focusing mechanism with a linear encoder, a reducer, a ball screw, a stepper motor, and a load. The functions of the ground test equipment are detailed in [29]. The electronic components involved in the drive system hardware are commonly used in aerospace design, and the main electronic components are shown in Table 1. The power consumption of the drive system is approximately 1.4 W. The length, width and height of the drive system hardware are 240 mm, 200 mm, and 42 mm, respectively. The mass of the drive system hardware is approximately 1.3 kg. The small size and light weight of the drive system hardware are very important for the space payloads. The linear encoder is employed to capture the information about the focal length and send it to FPGA. The reducer matches the speed and transmits the torque between the stepper motor and the ball screw. The main function of the ball screw is to convert the rotating motion into the linear motion, or to convert the torque into the axial repetitive force. The stepper motor can convert the electrical pulse signal into the corresponding angular displacement in order to achieve automatic control. Table 2 presents the main parameters of the stepper motor. The main electronic components presented in Table 1 and the motor shown in Table 2 both meet the stringent standards for aerospace applications. Next, the focusing results will be presented.

**Table 1:** Main electronic components.

Name	Model	Manufacturer
FPGA	A3PE3000-FG484I	ACTEL
Power MOSFET	JANSF2N7587U3	IR
Quadruple Differential Line Driver	JSR26C31AF	CETC
Quadruple Differential Line Receiver	JSR26C32F-S	CETC
Crystal Oscillator	ZA517	CASIC
Supervisory Circuit	MAX706TMJA	MAXIM
Transceiver	UT54ACS164245S	UTMC
Voltage Regulator	RHFL4913SCA-07V	ST
Power Module	PTH08T230WAD	TI

**Table 2:** Main parameters of the stepper motor.

Parameter	Value and Unit	Parameter	Value and Unit
Rated Voltage	28 V	Phase Inductance	85 mH
Phase Number	3	Phase Resistance	70 Ω
Rated Current	0.34 A	Moment of Inertia	$2.5\times 10^{-5}$ $\text{kg}\cdot {\text{m}}^{2}$
Polarity	Unipolarity	Step Angle	$1.8^{\circ }$

### Experimental results

**Fig 9:**
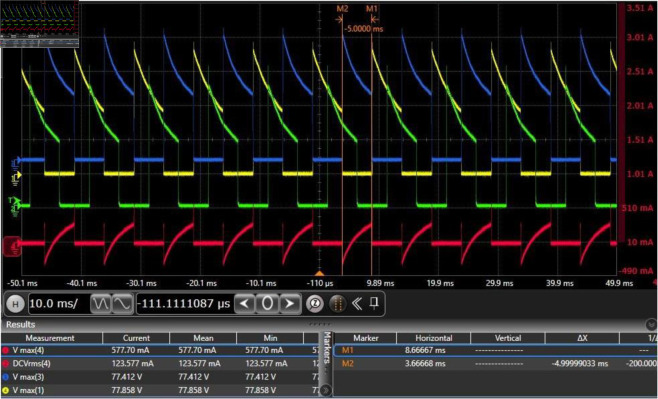
Drain potential waveform of the power MOSFET in each phase drive circuit and the current waveform of phase A, where the yellow line represents phase A, the green line represents phase B, the blue line represents phase C, and the red line represents the phase A current.

**Fig 10:**
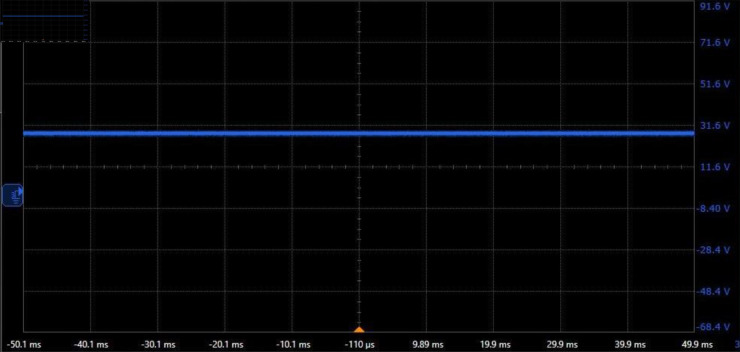
Waveform of $V_{d}$.

**Fig 11:**
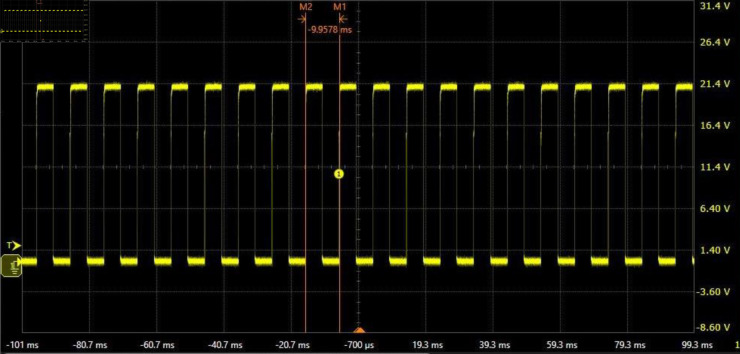
Collector potential waveform of the transistor in phase A drive circuit.

Before conducting the focusing experiment, the electrical performance of the designed motor drive circuit needs to be tested. The following parameter values are employed: $V_{c}=V_{d}=28$ V, *R*_*e*_ = 20 kΩ, and *R*_*f*_ = 1 Ω, the recommended values for other parameters are shown in Fig 4. In the absence of *D*_1_ and *D*_2_, the relevant potential and current waveforms are shown in Figs 9, 10, 11. In Fig 9, all drain potential waveforms are irregular rectangular waves. When the power MOSFET in phase *A* drive circuit is suddenly turned off, a higher induced electromotive force will be generated at the drain because there is no a current continuation loop. Although there is no the current continuation loop, the resulting high induced electromotive force still has a discharge loop because the power MOSFET in the turn-off state has a very small leakage current. The peak voltage of the induced electromotive force is about 77.8 V, and then gradually decays to about 38.4 V within 5 ms. During this period, the discharge current is very small (about 10 mA). This indicates that phase *A* has almost no the current during this period. In the presence of *D*_1_ and *D*_2_, there will be a large current in phase *A* when the power MOSFET is turned off. This will bring two results: first, phase *A* will produce a hindering torque, so the motor output torque will be reduced; second, phase *A* will produce unnecessary heat consumption, which will make the motor heat seriously. Therefore, the motor output torque is large in the absence of *D*_1_ and *D*_2_. The analysis results of the other two phases are similar to those of phase *A*. Fig 10 shows that $V_{d}$ is a constant voltage value (about 28 V). Fig 11 indicates that the collector potential waveform of the transistor in phase *A* drive circuit is a regular rectangular wave.

In fact, the drain potential waveform of the power MOSFET in each phase drive circuit is a regular rectangular wave in the presence of *D*_1_ and *D*_2_. Its pulse width is the same as the pulse width in the absence of *D*_1_ and *D*_2_. Its amplitude is about 28 V. In conclusion, the use of *D*_1_ and *D*_2_ in practical applications should follow the following principles:

- If the motor carries a light load, then in order to extend the service life of the switching device and improve the reliability of the circuit, the switching diodes *D*_1_ and *D*_2_ should be present.
- If the motor carries a heavy load, then in order to make the motor output a large torque and reduce the heat of the motor, the switching diodes *D*_1_ and *D*_2_ should be absent. However, the high-voltage resistant switching devices need to be selected to improve circuit reliability.

**Fig 12:**
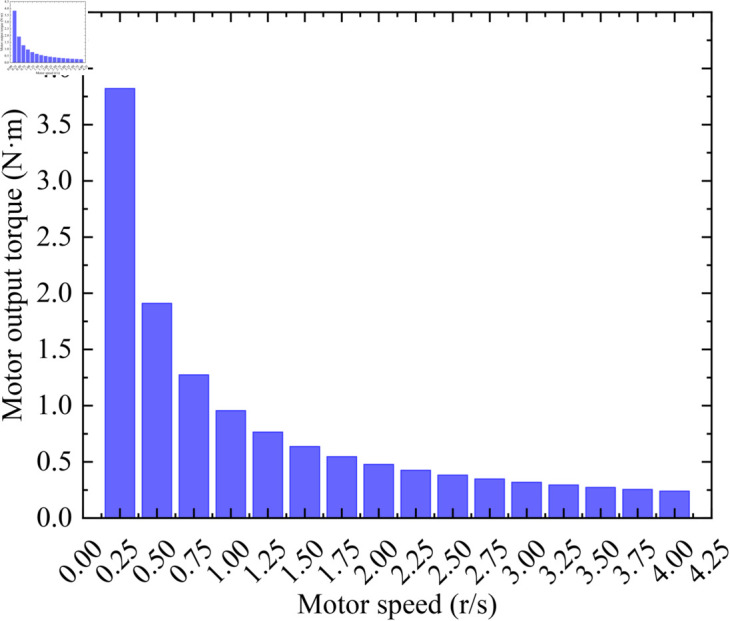
Motor output torque at different speeds.

**Fig 13:**
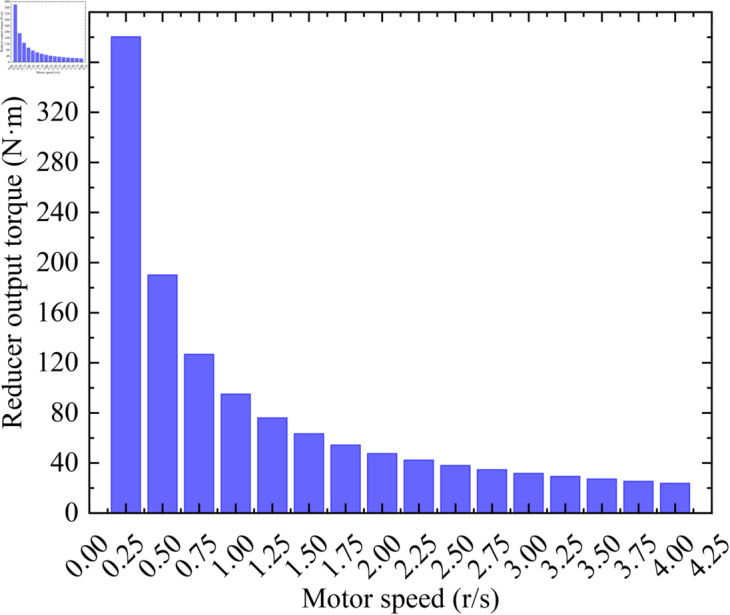
Reducer output torque at different speeds.

The switching diodes *D*_1_ and *D*_2_ are not employed due to the heavy payload of the satellite. This will enable the motor to output as much torque as possible to drive the heavy load. In order to quantitatively study the load-carrying capacity of the designed position monitoring and control system, the output torque of the motor and the output torque of the reducer at different speeds are presented in Figs 12 and 13, respectively. It can be seen from Figs 12 and 13 that the torque of both the motor and the reducer increases as the speed decreases, which is consistent with the conclusion derived from Eq (19). In addition, the large torque output of the reducer is very important for precise focusing. For example, when driving a heavy variable load, if there is not enough torque margin, the motor may miss steps due to being unable to carry the load, resulting in imprecise focusing. In summary, when focusing on a heavy load, if there are no requirements for focusing time, a lower motor speed can be selected to achieve greater torque. If fast focusing is needed, a higher speed should be chosen as much as possible while ensuring adequate torque. It is important to note that the motor speed cannot be too low, otherwise oscillations may occur, leading to inaccurate focusing.

To verify the performance of the designed drive system, the closed-loop focusing experiment and the open-loop focusing experiment are executed through the experimental setup shown in Fig 8, respectively. The following parameter values are employed: i=100:1, *L*_*l*_ = 2 mm, and *l*_*g*_ = 30 *μ*m. The range of motion of the focusing mechanism is ±6 mm and the focusing frequency is determined to be 400 Hz. The provisions are as follows: the forward direction is defined as the point “0” pointing to the point “ + 6 mm”, the reverse direction is defined as the point “0” pointing to the point “–6 mm”. For example, if *S*_*c*_>0,  + *S*_*c*_ means the position *S*_*c*_ in the forward direction, and −*S*_*c*_ means the position *S*_*c*_ in the reverse direction. If *S*_*c*_ = 0, it means the point “0”.

**Fig 14:**
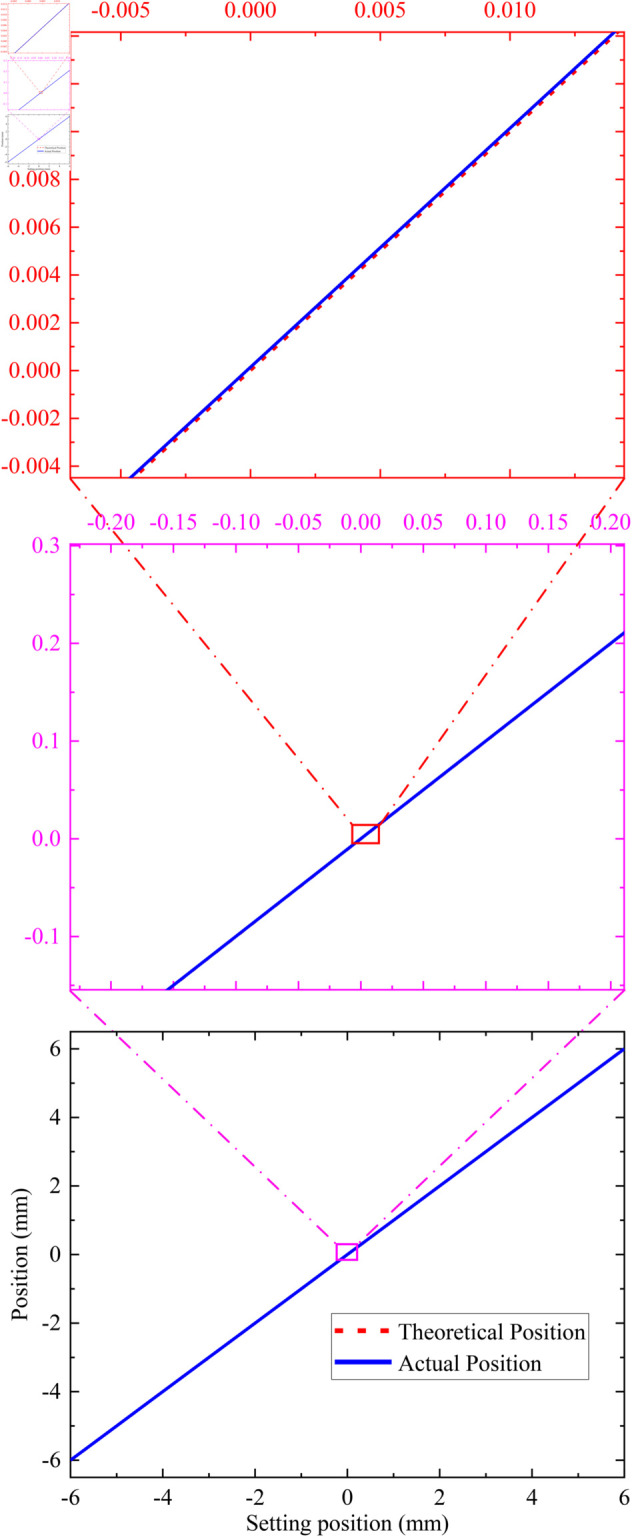
Graph of closed-loop focusing results.

**Fig 15:**
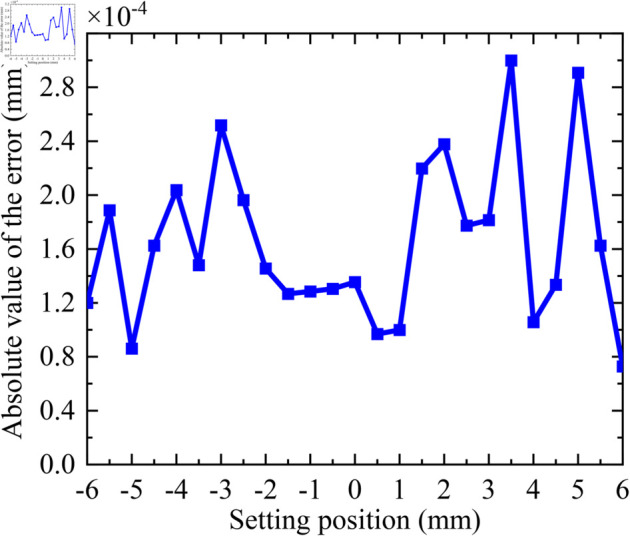
Graph of absolute value of closed-loop focusing error.

During the closed-loop experiment, the load is firstly adjusted from the point “0” to the point “–6 mm”, and then gradually adjusted from the point “–6 mm” to the point “ + 6 mm”, except for the point “–6 mm”, there is no change of direction during the whole process, and the final data results are shown in Table 3. The total time for closed-loop focusing is approximately 5 minutes. The closed-loop focusing results are shown in Fig 14. The absolute value of closed-loop focusing error is shown in Fig 15. From these experimental results, it can be seen that the closed-loop focusing function is normal, the load can be accurately adjusted to each target position, and the requirements can be met under the error threshold of ±0.0004 mm. Therefore, closed-loop focusing is suitable for scenarios requiring fine focusing.

**Table 3:** Experimental results of closed-loop focusing.

Setting Position (mm)	Actual Position (mm)	Absolute Value of the Error (mm)	Mean (mm)	Standard Deviation (mm)
–6	–6.00012	0.00012	$1.648\times 10^{-4}$	$6.03545\times 10^{-5}$
–5.5	–5.49981	0.00019		
–5	–4.99991	0.00009		
–4.5	–4.49984	0.00016		
–4	–3.99980	0.00020		
–3.5	–3.49985	0.00015		
–3	–2.99975	0.00025		
–2.5	–2.49980	0.00020		
–2	–1.99985	0.00015		
–1.5	–1.49987	0.00013		
–1	–0.99987	0.00013		
–0.5	–0.49987	0.00013		
0	0.00014	0.00014		
0.5	0.50010	0.00010		
1	1.00010	0.00010		
1.5	1.50022	0.00022		
2	2.00024	0.00024		
2.5	2.50018	0.00018		
3	3.00018	0.00018		
3.5	3.50030	0.00030		
4	4.00011	0.00011		
4.5	4.50013	0.00013		
5	5.00029	0.00029		
5.5	5.50016	0.00016		
6	6.00007	0.00007		

Note: The mean and standard deviation are both about “absolute value of the error”.

**Fig 16:**
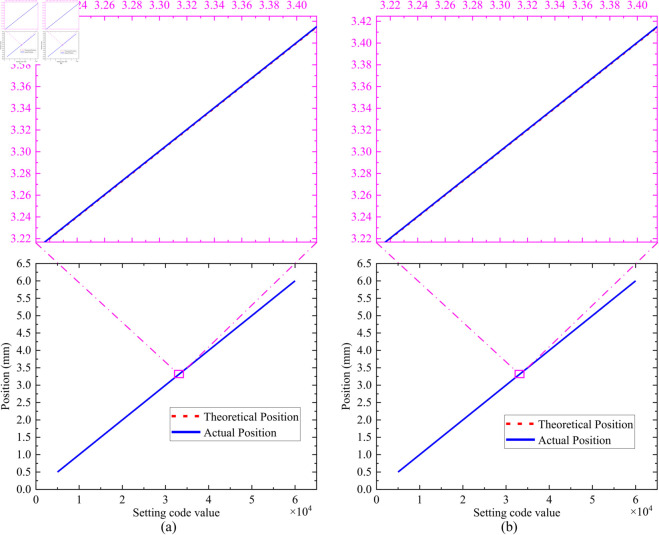
Graph of open-loop focusing results: (a) forward direction, (b) reverse direction.

**Fig 17:**
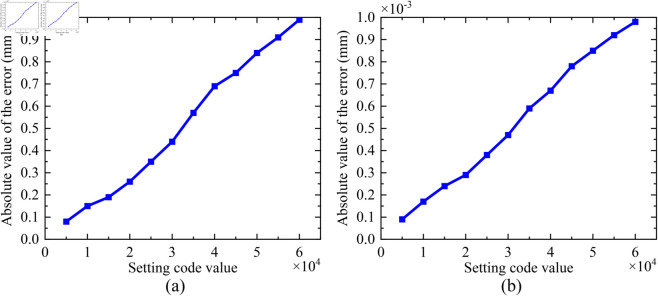
Graph of absolute value of open-loop focusing error: (a) forward direction, (b) reverse direction.

A threshold of 0~65535 is set for the number of the steps for the open-loop focusing. During the open-loop experiment, the focal position is first adjusted to the point “0”, and then the corresponding number of steps is adjusted, and the final data results are shown in Table 4. The total time for open-loop focusing is approximately 5 minutes. The open-loop focusing results are shown in Fig 16. The absolute value of open-loop focusing error is shown in Fig 17. From these experimental results, it can be seen that the open-loop focusing function is normal, the load can be adjusted to each target position. However, the focusing error is relatively large, and it increases with the number of the focusing steps. Therefore, open-loop focusing is suitable for scenarios requiring coarse focusing.

**Table 4:** Experimental results of open-loop focusing.

Motion Direction	Setting Code Value	Theoretical Position (mm)	Actual Position (mm)	Absolute Value of the Error (mm)	Mean (mm)	Standard Deviation (mm)
Forward Direction	5000	0.5	0.49992	0.00008	$5.18333\times 10^{-4}$	$3.1651\times 10^{-4}$
	10000	1	0.99985	0.00015		
	15000	1.5	1.50019	0.00019		
	20000	2	2.00026	0.00026		
	25000	2.5	2.50035	0.00035		
	30000	3	3.00044	0.00044		
	35000	3.5	3.50057	0.00057		
	40000	4	4.00069	0.00069		
	45000	4.5	4.50075	0.00075		
	50000	5	5.00084	0.00084		
	55000	5.5	5.50091	0.00091		
	60000	6	6.00099	0.00099		
Reverse Direction	5000	0.5	0.49991	0.00009	$5.35833\times 10^{-4}$	$3.06489\times 10^{-4}$
	10000	1	0.99983	0.00017		
	15000	1.5	1.50024	0.00024		
	20000	2	2.00029	0.00029		
	25000	2.5	2.50038	0.00038		
	30000	3	3.00047	0.00047		
	35000	3.5	3.50059	0.00059		
	40000	4	4.00067	0.00067		
	45000	4.5	4.50078	0.00078		
	50000	5	5.00085	0.00085		
	55000	5.5	5.50092	0.00092		
	60000	6	6.00098	0.00098		

Note: The mean and standard deviation are both about “absolute value of the error”.

It should be noted that the experimental conditions differ from the real working conditions in two main aspects. First, the experiment does not simulate a radiation environment. Second, the load used in the experiment is a simulated load rather than a real load. As mentioned earlier, the system’s radiation protection performance is ensured by using electronic components commonly employed in aerospace design. Due to the torque amplification effect of the reducer (Eq (19)) and the high output power of the designed electronic drive system, the output torque of the ball screw is sufficient to drive the real load that is heavier than the simulated load. Therefore, under these experimental conditions, the effectiveness of the proposed scheme can be verified.

The main limitation of the designed position monitoring and control system is that it should not operate under continuous power for extended periods. When *D*_1_ and *D*_2_ are present, prolonged operation of the proposed system can lead to severe overheating of the motor, resulting in a reduction of output torque. In severe cases, this can cause the motor to burn out and be unable to drive the load. Conversely, when *D*_1_ and *D*_2_ are absent, in view of the high peak voltage at the drain potential, long-term operation of the proposed system can shorten the lifespan of electronic components. In this study, the focusing operation is intermittent, with each working period typically not exceeding 10 minutes, followed by a rest period of about 5 minutes. Therefore, the aforementioned situations are almost impossible to occur.

From the above discussion, it can be inferred that the anti-irradiation performance of the designed position monitoring and control system is achieved by employing electronic components commonly used in aerospace design, as shown in Table 1. These electronic components are sourced from both domestic and international suppliers and have extensive experience in aerospace applications. Its strong load-carrying capacity and high positioning accuracy have been validated by experimental results. Considering that the designed system employs a constant voltage drive scheme, the control circuit becomes simpler compared to the constant current drive scheme presented in [30]. In addition, the proposed control scheme is also simpler than the neural network scheme for driving stepper motors mentioned in [31]. Therefore, the feasibility and practicality of the proposed scheme have been verified.

## Conclusion

A low-power position monitoring and control system for the focusing mechanism, specifically designed for aerospace applications, has been successfully developed and implemented. This system incorporates a constant voltage drive circuit tailored for a 3-phase, 4-wire stepper motor. Furthermore, an RS-422 transceiver circuit has been integrated to accurately capture data from a Renishaw linear encoder. Comprehensive focusing experiments were conducted, encompassing both closed-loop and open-loop configurations. The results indicate that the closed-loop focusing achieved an error of less than 0.0004 mm, while the open-loop focusing error incrementally increased with the number of focusing steps. The analysis of these experimental outcomes conclusively demonstrates the practicality and feasibility of the proposed scheme. This methodology holds promising potential for broader application to the stepper motor drive systems of other satellite-borne devices.

Because the designed system incorporates many high-grade and high-quality electronic components, the system cost increases. Under the premise that high system costs may limit practical application, the designed system still holds certain potential value and scalability, primarily reflected in the following aspects:

(1) **High-value, extremely high-precision fields:** For example, deep space exploration, astronomical telescopes, advanced laser communication, etc., which demand very high accuracy in positioning and focus adjustment. Traditional solutions are either costly or difficult to meet these requirements. If this system can significantly enhance focus accuracy and system stability, despite higher costs, it offers advantages in ensuring mission success and data quality, thus possessing considerable application potential.

(2) **Backup or supplementary solutions for critical missions:** Given the high cost, this system can serve as a high-end backup or supplementary solution in key tasks, ensuring the stability and reliability of core missions. In certain scientific research or national security projects, investing in a long-term, high-precision, low-maintenance monitoring system may be more cost-effective than frequent replacements or adjustments of traditional methods.

(3) **Technology verification and demonstration platform:** This system can act as a technological proof-of-concept platform for future more economical solutions. Validating system performance in high-value applications provides valuable experience for subsequent technological optimization and cost reduction, gradually promoting the system’s adoption in mid- to low-end applications.

(4) **Precision calibration and maintenance of high-end equipment:** In high-end optical devices, nuclear science experiments, extreme environment monitoring, and other fields, a precise position monitoring system can significantly improve calibration efficiency, reduce errors, and lower maintenance costs. Although initial investment is high, long-term use may yield higher efficiency and reliability benefits.

While high costs limit widespread adoption, the system still has broad application prospects in specialized fields where performance and reliability are critical. Future technological innovations, integration optimization, or mass production may lower costs, expand application scope, and enable broader deployment.
